# In-vehicle wireless driver breath alcohol detection system using a microheater integrated gas sensor based on Sn-doped CuO nanostructures

**DOI:** 10.1038/s41598-023-34313-6

**Published:** 2023-05-02

**Authors:** Hamid Reza Ansari, Zoheir Kordrostami, Ali Mirzaei

**Affiliations:** 1 Department of Electrical and Electronics Engineering, Shiraz University of Technology, Shiraz, Iran; 2 Research Center for Design and Fabrication of Advanced Electronic Devices, Shiraz University of Technology, Shiraz, Iran; 3 Department of Materials Science and Engineering, Shiraz University of Technology, Shiraz, Iran

**Keywords:** Electrical and electronic engineering, Nanoscale materials, Nanoscale materials

## Abstract

In this paper, we have developed an in-vehicle wireless driver breath alcohol detection (IDBAD) system based on Sn-doped CuO nanostructures. When the proposed system detects the ethanol trace in the driver`s exhaled breath, it can alarm and then prevents the car to be started and also sends the location of the car to the mobile phone. The sensor used in this system is a two-sided micro-heater integrated resistive ethanol gas sensor fabricated based on Sn-doped CuO nanostructures. Pristine and Sn-doped CuO nanostructures were synthesized as the sensing materials. The micro-heater is calibrated to provide the desired temperature by applying voltage. The results showed that by Sn-doping in CuO nanostructures, the sensor performance can be significantly improved. The proposed gas sensor has a fast response, good repeatability along with good selectivity that makes it suitable for being used in practical applications such as the proposed system.

## Introduction

Metal oxide gas sensors and their composites with other materials such as MXenes and metal chalcogenides, can detect different gases and vapors such as NO_2_, H_2_S, CO, C_2_H_5_OH, benzene, toluene, formaldehyde, using the resistance or voltage change that occurs as the result of their exposure to the target gases and vapors^1–12^. This resistance or voltage change then can be converted to sensing signal and if needed can be then converted from analog to digital to be processed. Thanks to low price and high performance of metal oxide gas sensors^2,13–17^, they can be integrated in different vehicles, smart electronic devices, exhaled breath analysis devices as well as the Internet of Things (IoT)^18–23^. Data communications are required to display the transmitted physical changes such as voltage to access the sensor output data. To this end, wireless data transmission reduces the size of the measuring devices, and makes them portable^4,24^. In recent years, the use of wireless communication platforms in gas sensor-based systems have provided many capabilities, including high speed data transmission, remote device control, and prevention of undesired events. In this context, development of highly sensitive, reliable and low-cost gas sensors with ability to communicate wirelessly is of importance. The IoT creates a network of interconnected devices that transmit information among themselves as well as to operators^13,25,26^.

Ethanol (C_2_H_5_OH), is mainly used as a solvent in various industries such as food, petrochemicals, medical applications and fuel industries. It is also used in alcoholic beverages and as a disinfectant. Therefore, it is important to develop technologies for ethanol gas sensing in car factories, food production, crude oil-based products, etc^27^. Along with many factors that lead to road accidents such as drowsiness, lack of safety and high speed, one of the most important factors is alcohol abuse by drivers^28–31^. In particular, metal oxide gas sensors can be used to detect ethanol gas^32–38^. Thus, they can be developed for the exhaled breath analysis of car drivers^39,40^ to ensure the health and alertness of the driver.

Copper oxide (CuO) is a p-type material with cost-effective synthesis, which has become an attractive choice for use in electronic and biocompatible devices due to its excellent electrical properties and long-term stability^41^. Previously, CuO in pristine or composite forms has been used for detection of ethanol^42–45^. However, this material as a gas sensor especially in pristine form has disadvantages such as low selectivity, high operating temperature and insensitivity to low concentrations of different gases^46,47^. This problem can be solved by using other materials in combination with CuO. For example, T_3_C_2_T_x_-CuO composite was used as NH_3_ sensor by Wang et al^48^. In particular, doping with other metals also can enhance overall performance of metal oxides^49^. n-type SnO_2_ is one of the most promising materials for sensor studies^50^, and its combination with other metal oxides can lead to more sensitive gas sensors^51,52^.

Motivated by above facts, in this research we used Sn-doping in CuO nanostructures to enhance gas sensing properties to a great extent. We have fabricated an in-vehicle wireless driver breath alcohol detection (IDBAD) system based on Sn-doped CuO nanostructures with fast response and short recovery time. The sensor can generate a signal to determine the concentration of the alcohol, then sends the vehicle location to the smartphone wirelessly and ultimately stops the vehicle from starting. The response of the proposed gas sensor exposed to 100 ppm of ethanol gas was 48 and its response and recovery times were 14 and 21 s, respectively. The proposed system had a power consumption of 1.6 Wh, which can be in standby mode for a long time due to its power supply by the car battery. Also, this system maintained 97% of the initial response even after 30 days, implying its long-term stability.

## Design and development of IDBAD system

One of the most important factors for the development and improvement of vehicles in the future is to save more lives from accidents. In this regard, the presence of alcohol gas sensors close to driver is essential to identify drunken drivers. So, we have developed a drunk driver detection system with the capability of wireless data transmission. The proposed system will be very useful when embedded in smart vehicles to prevent the drunk drivers from driving and to save the lives of many people.

**Figure 1:**
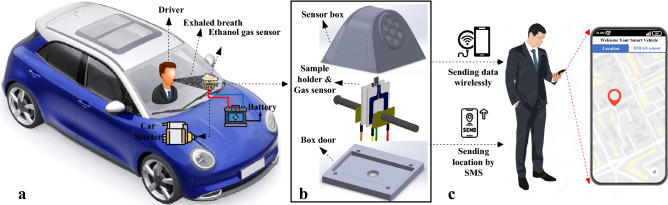
(**a**) The schematic of IDBAD system placed in the vehicle interior and its connections (drawn by Microsoft VISIO–version 2019 and flaticon.com). (**b**) A real image of the IDBAD system (drawn by SOLIDWORKS software—version 2018). (**c**) Display information received from the developed system on the smart phone (drawn by Microsoft VISIO—version 2019 and https://flaticon.com).

As shown in Fig. 1a, the IDBAD system can be placed in vehicle interior. The schematic of the gas sensor system to be mounted on the car dashboard can be seen in Fig. 1b. The black package has been fabricated by a 3D printer. The sensor and the holder position can be adjusted inside the box. As can be seen, there are several holes on the front of the box for exposure of the sensor to the driver's exhaled breath. A smartphone can be connected to the system via Wi-Fi (Fig. 1c). By placing an on/off switch between the developed system and the vehicle starter, the starter can be deactivated after the driver is identified as drunk. The proposed detection system is embedded in a small package with an aperture to allow the exposure of the gas sensor to the exhaled breath of car driver. The developed system consists of the gas sensor, a ceramic sample holder, a wireless communication module, a location transmittance module, a Wheatstone bridge circuit, a power supply circuit for gas sensor and modules, and an interface circuit for the connection between the Wheatstone bridge and the wireless communication module.

## Results and discussion

### Characterizations of sensing materials

**Figure 2:**
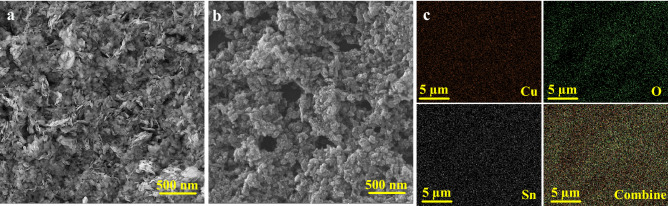
FE-SEM images of (**a**) pristine CuO nanostructures and (**b**) Sn-doped CuO nanostructures. (**c**) EDS elemental mapping of the Sn-doped CuO nanostructure.

Figure 2a presents the FE-SEM images of the synthesized pristine CuO sample, showing the morphology of the nanostructures. The size of the nanostructures in z direction is about 20–30 nm and the sizes in x and y directions are in the range of 500–700 nm. CuO nanostructures have a large surface area to volume ratio, providing a lot of absorption sites for incoming gas molecules. Figure 2b exhibits the FE-SEM image of the synthesized Sn-doped CuO nanostructures. Unlike pristine CuO nanostructures, the Sn-doped CuO nanostructures have a different surface morphology. They are composing of clusters of nanoparticles with sizes in the range of 20–30 nm. Figure 2c indicates the EDS elemental mapping analysis of Sn-doped CuO nanostructures. Clearly, Cu, O and Sn elements are uniformly distributed.

**Figure 3:**
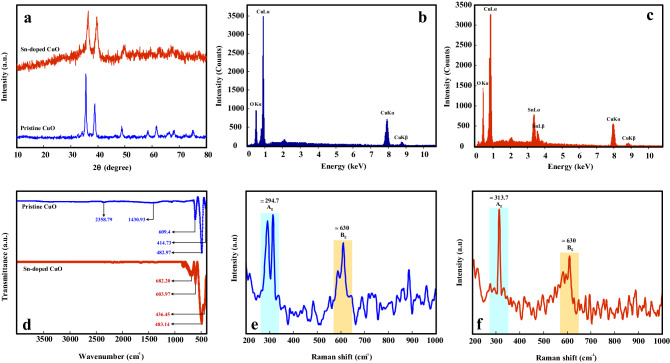
(**a**) XRD patterns of pristine and Sn-doped CuO nanostructures. EDS analysis results of (**b**) pristine CuO nanostructure and (**c**) Sn-doped CuO nanostructure. (**d**) FTIR spectra of pristine and Sn-doped CuO nanostructures. Raman spectra for (**e**) pristine CuO nanostructure and (**f**) Sn-doped CuO nanostructure.

Figure 3a shows X-ray diffraction (XRD) patterns of pristine and Sn-doped CuO nanostructures. The diffraction peaks of pristine CuO nanostructure are sharp, showing the high crystallinity of the synthesized sample. The peaks located at 35.5, 38.6, 48.6, 58.1, 61.4, 66.0 and 67.9° are respectively, belong to $\left( {\overline{1}11} \right)$, (111), $\left( {\overline{2}01} \right)$, (202), $\left( {\overline{1}13} \right)$, $\left( {\overline{3}11} \right)$ and (113) crystalline planes of monoclinic crystalline CuO (JCPDS card no. 89-2530)^53^. For Sn-doped sample, no additional peaks related to other phases was observed, demonstrating the incorporation of Sn into CuO lattice^54,55^.

EDS elemental analysis results for pristine and Sn-doped CuO nanostructures are presented in Fig. 3b and c, respectively. For pristine sample, the peaks related to Cu and O elements were detected. However, after Sn-doping an additional peak related to Sn was observed, confirming doping of Sn into CuO lattice.

Fourier-transform infrared spectroscopy (FTIR) analysis results for pristine and Sn-doped CuO nanostructures are presented in Fig. 3d. In the FTIR spectrum of pristine CuO nanostructures, three peaks at 414.73 cm^−1^, 482.97 cm^−1^ and 609.4 cm^−1^ can be seen that are belong to the lattice vibrational modes of CuO. Also, the weak peaks at 1430.93 cm^−1^ and 2358.79 cm^−1^ are related to the vibration of C–H bonds. The peaks related to O–H bonds are not observed, indicating complete dryness of the sample^56^. For Sn-doped sample, the main peaks at 436.45, 483.14, 603.97 and 682.2 can be related to the vibration modes of Sn–O and Cu–O.

Raman spectra of pristine and Sn-doped CuO nanostructures are presented in Fig. 3e and f, respectively. Both Raman spectra exhibited two distinct peaks at around 294.7 and 630 cm^−1^, which are respectively assigned to the A_g_ and B_g_ vibration modes of CuO^57^.

### Gas sensing measurement

**Figure 4:**
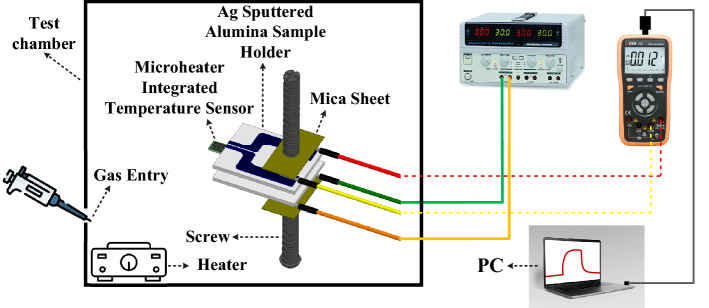
The gas sensing setup.

The static method was used to measure the response of the gas sensors. The microsyringe injected a desirable amount of target gas into the gas chamber, which was mixed with the air. For liquids such as VOCs, the liquid was heated on a heating plate until completely evaporated and mixed. Following formula was used to calculate the concentration of VOCs in gas chamber:

1 $$V(\mu L)=\frac{VCM ({T}_{stan}){10}^{-3}}{22.4\uprho\,({T}_{sh})}$$

where V (μL) is the volume of VOC, V (L) is the volume of the test chamber, C (ppm) is the concentration of gas, M is the molar weight of VOC, Tstan (K) is the temperature of standard condition, Tsh (K) is the ambient temperature, ρ (g cm^−3^) is the density of VOC, and 22.4 is the molar volume of the standard gas. For gases such as H_2_S, NH_3_ and CO_2_, dry air was used as a balance gas. Dry air-balanced target gases from cylinders and dry air without humidity were introduced into the gas chamber using mass flow controllers (MFCs). The response was defined as V_g_/V_a_ where V_a_ and V_g_ are the voltages in air and in the presence of target gas, respectively. The response time and recovery time, were defined as the period of time needed for the sensor voltage to return to 90% of its final stable value^58^. The schematic diagram of the gas sensing procedure is shown in Fig. 4.

### Gas sensing studies

Figure S1a shows the variations of the baseline resistance of pristine and Sn-doped CuO gas sensors as a function of temperature. In both cases, the resistance decreases upon increase of the temperature, showing semiconducting behavior of the gas sensors^59^. Also, at all temperatures, the baseline resistance of Sn-doped CuO gas sensor is higher than that of pristine CuO gas sensor. This increase in the resistance is due to the presence of Sn ions in the CuO lattice, which play the role of electron donor. As a result, the density of holes will decrease and then the baseline resistance will increase. Besides, adding Sn to the p-type CuO will result in formation of a depletion area and then a potential barrier, which can be another reason for increasing the baseline resistance. Good resistance stability of Sn-doped gas sensor at different temperatures is shown in Fig. S1b. It is shown that the values of the resistance of the gas sensor upon increasing and decreasing of the sensing temperature are almost the same, revealing the good resistance stability of the gas sensor. Also, Fig. S1c shows good stability of the sensor resistance at a fixed temperature for a long time of more than 1600 s. Overall, the Sn-doped gas sensor shows a stable resistance that is beneficial for sensing studies.

**Figure 5:**
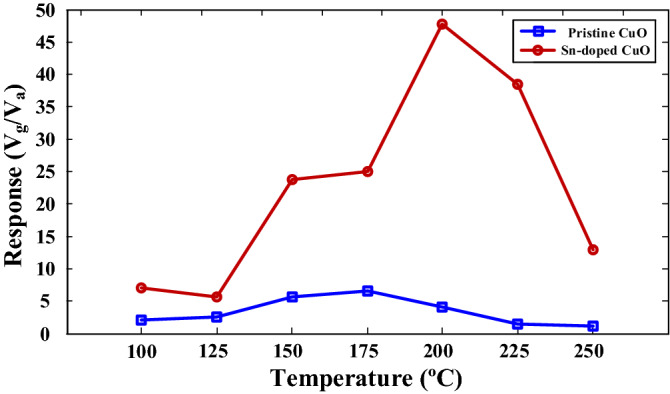
Response of pristine and Sn-doped CuO gas sensors to 100 ppm ethanol at different temperatures.

To determine the optimal working temperature of the gas sensors, they were exposed to 100 ppm ethanol gas at different temperatures (Fig. 5). In both sensors, initially the response increased with temperature, then reached to a maximum value and finally decreased. The reason for this behavior is that at low temperatures there is no enough energy for gas to overcome the absorption barrier energy and at high temperatures the desorption rate is higher than absorption rate. At optimal sensing temperature, the absorption rate is equal to desorption rate and the maximum response is observed. The maximum response of pristine gas sensor to 100 ppm ethanol is 5.1 at 175 °C and it is increased to 48 for Sn-doped CuO gas sensor at 200 °C, demonstrating the promising effect of Sn-doping in CuO for ethanol gas sensing.

**Figure 6:**
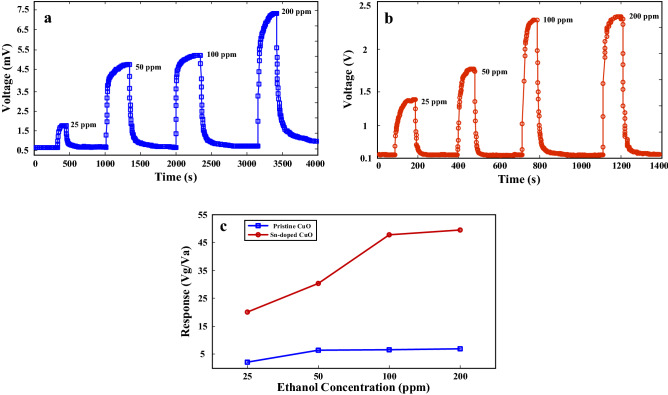
Dynamic voltage curves of (**a**) pristine CuO gas sensor and (**b**) Sn-doped CuO gas sensor to 25–100 ppm ethanol gas. The testes were performed at their optimal sensing temperatures. (**c**) Calibration curves of gas sensors at their optimal working temperature.

We also exposed both gas sensors to different concentrations of ethanol at their optimal sensing temperature. Figure 6a and b show the dynamic voltage changes of pristine and Sn-doped CuO gas sensors to 25–200 ppm ethanol gas at 175 and 200 °C, respectively. In both cases, the sensors showed p-type behavior, resulting from intrinsic p-type nature of CuO. Corresponding calibration curves of both gas sensors are presented in Fig. 6c. Obviously for all tested ethanol concentrations (25–200 ppm), the response of Sn-doped gas sensor is higher than that of pristine gas sensor.

**Figure 7:**
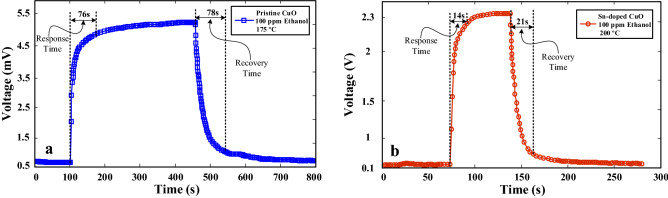
Dynamic voltage curves of (**a**) pristine CuO gas sensor and (**b**) Sn-doped CuO gas sensor to 100 ppm ethanol gas and at their optimal sensing temperatures.

Figure 7a and b show the dynamic voltage curves of pristine and Sn-doped gas sensors to 100 ppm ethanol at their optimal sensing temperatures, respectively. Based on these curves, the response time and recovery time for pristine gas sensor were calculated to be 76 and 78 s, respectively. Also, the response time and recovery time of Sn-doped gas sensor were 14 and 21 s, respectively. These values show the faster dynamics of Sn-doped gas sensor. Also, Fig. S2 shows the dynamic voltage curves of both gas sensors to various concentrations of ethanol gas, indicating response time and recovery time of both gas sensors to 25–200 ppm ethanol gas. The results show that the Sn-doped gas sensor is superior to pristine gas sensor not only due to its higher response, but also due to its faster dynamics. Doping of CuO provides a space charge area that helps in increased modulation of the resistance in the presence of gas. Sn-doping in CuO also leads to more porosity and an increase in the surface to volume ratio. More porosity in the gas-sensitive layer causes more gas penetration in the material structure, which will result in more interaction of Sn-doped CuO nanostructures with ethanol gas that eventually improves the sensor response.

**Figure 8:**
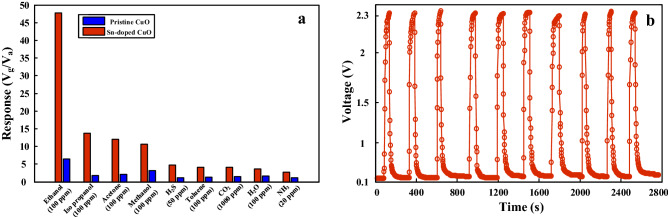
(**a**) Selectivity histogram of pristine and Sn-doped gas sensors to various concentrations of different gases. (**b**) Repeatability of the Sn-doped gas sensor to 100 ppm ethanol at 200 °C.

Selectivity is one of the most important features of a gas sensor. In fact, weak selectivity leads to false alarm which means that the sensor is unable to detect the target gas. To explore the selectivity of both gas sensors, they were exposed to various concentrations of different gases at their optimal working temperatures (Fig. 8a). Obviously, the Sn-doped gas sensor shows a very high response to ethanol gas and much lower response to other gases such as acetone, isopropanol, methanol, H_2_S, toluene, CO_2_, and NH_3_. Even though the pristine gas sensor also shows its highest response to ethanol gas, its response to other gases is close to that of ethanol gas, showing the poor selectivity of this gas sensor. Interestingly, the Sn-doped gas sensor also showed a low response to water vapor, which is present in exhaled breath of drunk car drivers. One of the important requirements for gas sensors to be used in a smart system is repeatability. The repeatability of Sn-doped gas sensor was studied by exposing of this gas sensor to 100 ppm ethanol gas during ten sequential cycles (Fig. 8b). There is a negligible difference between the sensing behaviors in different cycles, demonstrating the excellent repeatability of the Sn-doped gas sensor.

**Figure 9:**
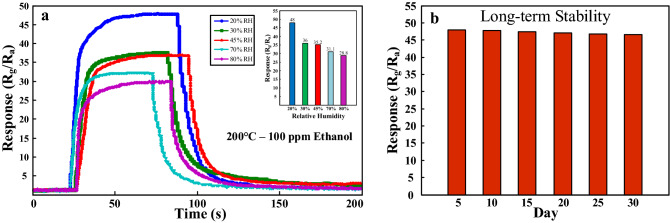
(**a**) Dynamic responses of Sn-doped CuO gas sensor to 100 ppm ethanol at 200 °C in the presence of various RH levels. Inset shows variations of the response versus the RH levels. (**b**) Long-term stability of the Sn-doped CuO gas sensor.

We also studied the sensing behavior of the Sn-doped gas sensor in the presence of 20–80% relative humidity (RH), measured by a humidity sensor. Figure 9a indicates the dynamic responses of the Sn-doped CuO gas sensor to 100 ppm ethanol gas in the presence of different percentages of RH at 200 °C. Also, as shown in inset of Fig. 9a, the response of the sensor in the presence of 20, 30, 45, 70 and 80% RH is 48, 36, 35.2, 31.1 and 28.8, respectively. In general, with increasing of the RH, more water molecules become adsorbed on the surface of sensor, resulting in decrease of the available adsorption sites. Hence, less amounts of ethanol molecules can be adsorbed on the surface of the gas senor, resulting in a decrease of the response in the presence of humidity. However, it should be noted that still in the presence of 80% RH, the sensor has a high response to ethanol gas. The stability of the Sn-doped CuO gas sensor was examined during one month with 5-days intervals (Fig. 9b). There were almost no variations of the response and even after one month the response was 97% of its fresh state. This confirms the high stability of the sensor.

The ethanol sensing performance of the Sn-doped CuO gas sensor in this research is compared with those of other gas sensors in Table 1. Obviously, it shows much higher response compared with other gas sensors. Furthermore, its sensing temperature is also relatively low. Accordingly, it can be considered as a very promising sensor for detection of ethanol gas.

**Table 1:** Comparison of the ethanol gas sensing properties of the Sn-doped CuO gas sensor with those of other gas sensors.

Sensing material	T (°C)	Conc. (ppm)	Response (R_a_/R_g_) or (R_g_/R_a_)	Refs.
Co-doped SnO_2_ nanobelts	300	200	49.4	^60^
SnO_2_@POMs@WO_3_ nanofibers	280	100	8.8	^61^
TiO_2_/SnO_2_ films	260	100	10	^62^
Flower-like Sn/SnO_2_	250	1000	17.46	^63^
SnO_2_ nanoparticles-NiO nanocuboids heterojunctions	250	100	28	^64^
CuO/rGO nanosheets	175	100	10.54	^65^
Co_3_O_4_ nanorods	150	100	21.46	^66^
Co_3_O_4_-ZnSnO_3_ nanowires	300	100	5.57	^67^
Co_9_S_8_ nanotubes	160	100	7.326	^68^
In_2_O_3_ nanowires	25	500	1.3	^69^
MoS_2_–NiCo_2_O_4_ nanocomposite	170	100	9	^70^
Sn-doped CuO nanostructures	200	100	48	This work

In gas sensors based on metal oxides, changes in resistance, current or voltage can be tracked to monitor the concentration of a target gas. In this paper, we converted the resistance change to the voltage change and tracked the voltage change as the sensor output. Changes in voltage values are directly dependent on the concentration of target gas in surrounding atmosphere. In air, the oxygen molecules interact with the surface of the gas-sensitive layer and form ${\text{O}}_{2}^{ - }$ and O^−^ species as follows:

2 $${\text{O}}_{{2\left( {{\text{gas}}} \right)}} \to {\text{O}}_{{2\left( {{\text{ads}}} \right)}}$$

3 $$O_{{2\left( {{\text{ads}}} \right)}} + e^{ - } \to O_{{2\left( {{\text{ads}}} \right)}}^{ - }$$

4 $$O_{2}^{ - } \left( {{\text{ads}}} \right) + e^{ - } \to O^{ - }$$

**Figure 10:**
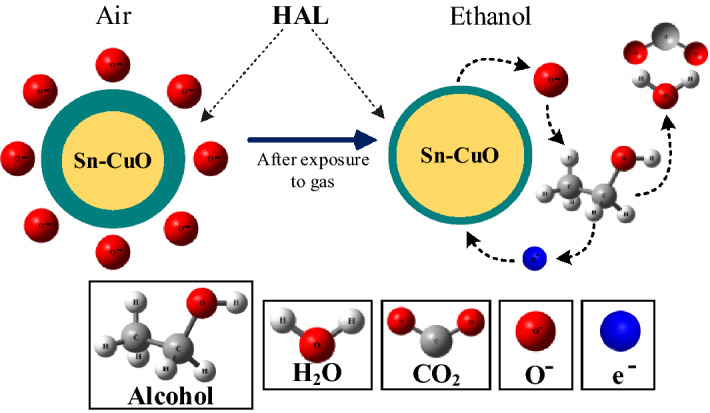
Schematic illustration of ethanol sensing mechanism in Sn-doped CuO nanostructures.

As a result of the absorption of electrons by oxygen ions, the concentration of holes on the surface of Sn-doped CuO increases and a hole accumulation layer (HAL) is formed (Fig. 10; left), leading to a decrease of resistance in air relative to vacuum condition. When the sensor is exposed to ethanol gas, it reacts with already adsorbed oxygen species and electrons will be released:

5 $$C_{2} H_{5} {\text{OH + }}O^{ - } \to CO_{2} + H_{2} O + e^{ - }$$

Upon release of electrons, the concentration of holes and the width of the HAL decrease (Fig. 10; right) which results in a sharp increase in voltage.

Furthermore, upon doping of Sn into CuO lattice, some structural defects can be created which eventually provide the preferential adsorption sites for ethanol gas molecules and contribute to response enhancement in Sn-doped gas sensor^42^.

### Details of designed system

**Figure 11:**
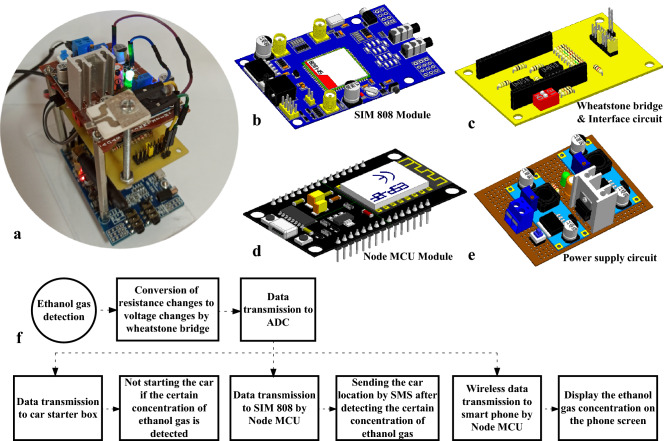
(**a**) Digital photo of IDBAD system. (**b**) Schematic of SIM808 module. (**c**) Schematic of designed Wheatstone bridge and interface circuits. (**d**) Schematic of Node MCU. (**e**) Schematic of the power supply circuit of the entire system. [(**b**)–(**e**) were drawn by HFSS software—version 2015] (**f**) Block diagram of IDBAD system working mechanism.

Figure 11a shows a real photograph of the fabricated sensing device. The developed system consists of three boards that are placed sequentially on top of each other. A quad-band GSM/GPRS module (SIM808) was used to collect and send the location data that can be seen on the first floor of the system in Fig. 11b. A GPS antenna with a frequency of 1575.42 MHz and a supply voltage of 3–5 V was used to send the location. Also, a GSM antenna with 2 dB antenna gain was used to send the SMS. A step-down (buck) switching regulator (LM 2596) was used to reduce the 12 V battery voltage to 9 V for powering the GSM/GPRS board.

Figure 11c and d illustrate the designed and fabricated printed circuit board (placed on the second floor of the developed system) which includes a Wheatstone bridge circuit, an IoT platform (Node-MCU module), and a circuit that interfaces the Wheatstone bridge to the Node-MCU. The Node-MCU provides a wireless communication link between the gas sensor and the smart phones or monitoring computer connected to the internet. A Wheatstone bridge circuit including 1 kΩ, 2.2 MΩ and 2.2 MΩ resistors was used to convert the gas sensor resistance change to the voltage change. The output voltage is generated between points B and D of the Wheatstone bridge (Fig. S3a) to subtract the voltages from each other (point B and D), an interface circuit was designed based on two operational amplifiers (Fig. 11c).

Generally, it is the resistance variations that are tracked in metal oxide gas sensors^71^. However, by using a Wheatstone bridge, we converted the resistance change to the voltage change so that it can be used for next step processes. This conversion also reduces the errors caused by the battery and the next stage circuit^72–74^. That is because the sensor resistance value in this design, has no dependence on the internal resistance of the battery or the interface circuit which may reduce the accuracy of the measurements. IDBAD system is powered by connecting to the vehicle battery. Since a typical car battery can provide 12 V voltage and up to 60 A current, there will be no problem in supplying the system, when the vehicle is working. Given that the power consumption of the sensor is 1.6 Wh, when the vehicle is switched off, it can remain in standby mode for about 19 days and there is no need to insert a battery to store the energy in the proposed system. Thus, the proposed system can recognize the ethanol gas for a long-time without external power sources and manage appropriate safety measures. The integrated micro-heater design in the proposed gas sensor has a lower energy consumption in comparison to other heater types used in similar gas sensors. This is very important since in general the working temperatures of metal oxide gas sensors are high, leading to high energy consumption and need for external heater on the back side of the gas sensor^75^.

The Wheatstone bridge was designed to work in four different operating modes. Each operating mode can be selected by placing the jumpers in the appropriate position (Fig. S3b). This capability was added to the circuit to improve the ADC resolution. In this way, different resistance ranges are supported without losing the ADC accuracy. For this purpose, four Wheatstone bridges were designed using 2.2 MΩ, 1 MΩ, 100 kΩ and 10 kΩ resistors (two of each resistor in each bridge). Two other sides of the bridges had the variable resistance of the sensor and a 1 kΩ resistor. Hence, testing of a wide range of alcohol concentrations is possible.

Finally, the analog voltage at the output of the interface circuit was converted to digital voltage by the ADC of Node-MCU, which can then wirelessly send the data to the Android app installed in the phone (Fig. 11d), by using the Wi-Fi microchip (ESP-8266). The voltage required to power the Node-MCU was 5 V, which was supplied through the micro-USB port, and its current consumption is only 80 mA. Whenever the output voltage of the detection system reached to the voltage value corresponding to a certain concentration of ethanol gas (already programmed in the microcontroller), the location of the vehicle was automatically sent by SMS to the destination phone number (already programmed).

The upper floor of the IDBAD system (Fig. 11e) was related to supplying the entire system. This board was consisted of two step-down (buck) switching regulators (LM 2596) and a power supply circuit including a L7805 regulator to supply Node-MCU, a heat sink, a capacitor (470 µF), a resistor (2.2 kΩ) and a green LED. Two LM 2596 modules were set at 9 and 4.1 V voltages and were used to power the SIM808 and supply the micro-heater that controlled the operating temperature of the sensor, respectively. On the power supply board input terminal was connected to the vehicle battery and there was a push button next to the terminal to on/off the system. The block diagram shown in Fig. 11f exhibits the working mechanism of the IDBAD system. The details of the designed Wheatstone bridge circuit and the interface circuit are shown in Figs. S3c–S3f.

**Figure 12:**
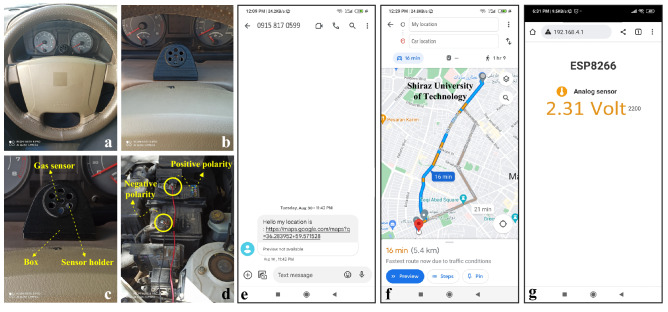
(**a**–**c**) Digital photos of gas sensor for practical application inside of the vehicle. (**d**) Connecting the device to the car battery. (**e**) Sending location to smart phone by SIM 808 module. (**f**) Display the direction from the origin to the destination on the Google Map. (**g**) Real-time display of voltage changes on the smart phone [(**e**–**g**) were screenshot from the smartphone].

As presented in Fig. 12a–g, in practice, the sensor can rapidly identify drunk drivers, generating a signal, sending the vehicle location to the smartphone and prevent the vehicle from starting.

## Conclusions

In this paper, we introduced a location-sender IDBAD gas sensing system based on Sn-doped CuO nanostructures. The proposed sensor was highly sensitive to alcohol exposure. The fabricated gas sensor was easily incorporated into a vehicle for alcohol detection. When the alcohol was sensed by sensor, the communication system wirelessly sent the vehicle location and stopped the vehicle. The developed IDBAD system showed very fast response and recovery time along with good selectivity to ethanol gas. In developed system, power can be supplied to the gas sensor from vehicle battery and also due to relatively low working temperature of gas sensor there is no concern about lack of power supply to the developed IDBAD system. We successfully used our proposed system in a real application and therefore we believe that it can be used for practical applications in different vehicles easily.

## Methods

### Starting materials

Analytical grades of copper (II) acetate monohydrate (C_4_H_8_CuO_5_, 99%; Merck), tin (IV) chloride pentahydrate (SnCl_4_·5H_2_O, 98%; Exir), sodium hydroxide (NaOH, 98%; Merck) and ethyl alcohol (C_2_H_6_O, Absolute; Merck) were used without extra purification.

### Synthesis of pristine and Sn-doped CuO nanostructures

We used a chemical precipitation technique to synthesize the pristine and Sn-doped CuO nanostructures. In a typical procedure, 1996 mg C_4_H_8_CuO_5_ was dissolved in 50 mL DI water under vigorous stirring at room temperature (15 min) to form a homogeneous solution. Subsequently, 50 mL NaOH (600 mM) aqueous solution was added dropwise into the above solution. The obtained solution was kept under continuous stirring at 60 °C for 1 h until the solution turns black. Next, the products were washed by centrifuge at 2500 rpm with ethanol and DI water to remove impurities. This procedure was repeated for four times. Then the powders were dried at 80 °C and the remaining water and ethanol were evaporated. The final powders were annealed at 400 °C for 2 h in muffle furnace to obtain CuO nanostructures.

For synthesis of Sn-doped CuO nanostructures, 20 mL SnCl_4_·5H_2_O (100 mM) aqueous solution was slowly added to 200 mM C_4_H_8_CuO_5_ solution under continuous stirring. The rest of the procedure was similar to that already explained for synthesis of pristine CuO nanostructures. The schematic and block diagram of different steps used for the synthesis of pristine and Sn-doped CuO nanostructures are presented in Figs. S4a and b, respectively.

### Characterizations

The morphological analyses of sensing material were performed using field emission scanning electron microscopy (FE-SEM, MIRA3-TESCAN-XMU, Czech Republic). X-ray energy dispersive spectrometry (EDS) analysis was used to study chemical composition of samples. The phase and crystal structure studies were carried out using X-ray diffraction (XRD, Bruker D8-Advanced X-ray diffractometer, Germany) with Cuk_α_ radiation (λ = 1.5409 Å). Fourier-transform infrared spectroscopy (FTIR, Bruker-Tensor II, Germany) was used for identification of chemical compounds in the products. Raman spectra were recorded using Horiba, XploRA PLUS, France, with a laser excitation wavelength of 532 nm.

### Gas sensor fabrication

**Figure 13:**
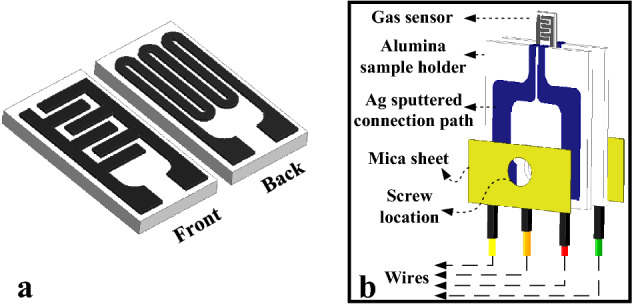
(**a**) Front and back sides of sensor substrate. (**b**) Schematic of sensor holder.

As shown in Fig. 13a, an alumina (Al_2_O_3_) substrate (6 × 3 × 0.5 mm^3^) was equipped with Pt interdigitated electrodes (pitch of 150 µm) on the front side. Its backside was used for integration of a Pt micro-heater. The synthesized sensing materials were ultrasonicated with DI water to create a homogeneous solution. Sensing material was coated over the substrate via drop casting technique (3 µl) and then it was heated at 50 °C to dry completely. Finally, it was annealed at 400 °C for 30 min to remove water and dry completely. Since the sensor pads were very small and thin, a sample holder (Fig. 13b) was designed to provide promising electrical connections to the sensor. It was comprised of two alumina plates, gas sensor, four wires and two mica sheets. The silver connection path to the sensor pads was over the alumina plates that were used as large conductor substrates. Silver pads with thicknesses of 500 nm were deposited on the alumina plates by employing DC-spattering and a shadow mask. The silver pads were connected to small sensor pads from one end and the copper wires from the other end. By screwing two mica sheets, the silver pads of the two alumina plates were connected to the sensor pads and to the heater pads and the copper wires were hold tight. By applying an external voltage to Pt micro-heater and because of the Joule effect the heater generated heat, leading to increase of sensing device.

## Supplementary Information

Supplementary Figures.

## Data Availability

The datasets generated and analyzed during the current study are available from the corresponding author on reasonable request.
