# Supplementary material for: In-vehicle wireless driver breath alcohol detection system using a microheater integrated gas sensor based on Sn-doped CuO nanostructures
Source: Sci Rep. 2023 May 2;13:7136. doi: 10.1038/s41598-023-34313-6 (PMC10154331; doi:10.1038/s41598-023-34313-6)
Supplement: Supplementary file 1 — Supplementary Figures. [file 41598_2023_34313_MOESM1_ESM.docx]

**Supporting Information**

**In-Vehicle Wireless Driver Breath Alcohol Detection System Using a Microheater Integrated Gas Sensor Based on Sn-Doped CuO Nanostructures**

Hamid Reza Ansari^1,2^, Zoheir Kordrostami^1,2,*^, Ali Mirzaei^3^

^1^ Department of Electrical and Electronics Engineering, Shiraz University of Technology, Shiraz, Iran

^2^ Research Center for Design and Fabrication of Advanced Electronic Devices, Shiraz University of Technology, Shiraz, Iran

^3^ Department of Materials Science and Engineering, Shiraz University of Technology, Shiraz, Iran

Corresponding Author`s e-mail: [kordrostami@sutech.ac.ir](mailto:kordrostami@sutech.ac.ir)

**Figure S1.** (**a**) Variations of the resistance versus sensing temperature for pristine and Sn-doped CuO gas sensors. (**b**) Resistance changes of the Sn-doped CuO gas sensor upon increase and decrease of sensing temperature. (**c**) Resistance stability of the pristine and Sn-doped CuO gas sensors at their working temperatures.

**Figure S2.**  Dynamic voltage curves of pristine gas sensor to (**a**) 25, (**b**) 50 and (**c**) 200 ppm ethanol gas at 175°C. Dynamic voltage curves of Sn-doped CuO gas sensor to (**d**) 25, (**e**) 50 and (**f**) 200 ppm ethanol gas at 200°C.

**Figure S3.** (**a**) Wheatstone bridge circuit. (**b**) jumpers on the Wheatstone bridge circuit (drawn by HFSS software – version 2015). (**c**) Wireless connection unit. (**d**) Wheatstone bridge voltage range change unit. (**e**) Interface circuit unit. (**f**) PCB of Wheatstone bridge and interface circuits (drawn by Altium Designer software – version 2019).

**Figure S4**. (**a**) Schematic illustration of the different steps used for synthesis of pristine and Sn-doped CuO nanostructures. (**b**) Block diagram of the synthesis of pristine and Sn-doped CuO gas sensors.
